# Rare presentation of double-clonal Waldenström macroglobulinemia with pulmonary embolism: A case report

**DOI:** 10.1515/biol-2022-0619

**Published:** 2023-06-14

**Authors:** Yan Sun, Fan-Jun Meng, Jun-Xia Huang, Xue-Shen Yan, Xia Zhao, Jing-Jing Zhou, Yan Gao

**Affiliations:** Department of Hematology, The Affiliated Hospital of Qingdao University, No. 1677 of Wutaishan Road, Huangdao District, Qingdao 266000, Shandong, China

**Keywords:** case report, double-clone, lymphoplasmacytic lymphoma, pulmonary embolism, Waldenström macroglobulinemia

## Abstract

Waldenström macroglobulinemia (WM) rarely leads to pulmonary embolism. Due to its low incidence, the underlying pathophysiology, prognosis, and optimal treatment remain largely unexplored and uninvestigated. In this study, a patient with a double-clonal WM, a rare subtype, presented with pulmonary embolism. The patient had a small number of plasma cells without morphological abnormalities, and an effective therapeutic response was observed. Nonetheless, the clinical prognosis requires a long-term follow-up.

## 1 Introduction

Lymphoplasmacytic lymphoma (LPL)/Waldenström macroglobulinemia (WM) is a type of small B-cell indolent lymphoma with plasma cell differentiation, featuring a broad range of signs and symptoms arising from markedly elevated serum levels of monoclonal immunoglobulin M (IgM). The majority of patients diagnosed with this disease are elderly and typically present with symptoms such as anemia, hemorrhage, and hyperviscosity syndrome. This specific type of non-Hodgkin’s lymphoma (NHL) is relatively rare, accounting for less than 2% of NHLs. While the increase in serum IgM is monoclonal in most patients, it may be polyclonal in a very small fraction of WM patients. Among WM patients, pulmonary complications and infections are the most common causes for death, with pulmonary embolism being a much rarer complication.

In this study, a 62-year-old male, with pulmonary infection and embolism, was eventually diagnosed with double-clonal WM. Pulmonary embolism may be the initial manifestation of WM, highlighting the importance of an underlying gammopathy in older patients presenting with pulmonary embolism.

## 2 Case presentation

### 2.1 History of the present illness

**Figure 1:**
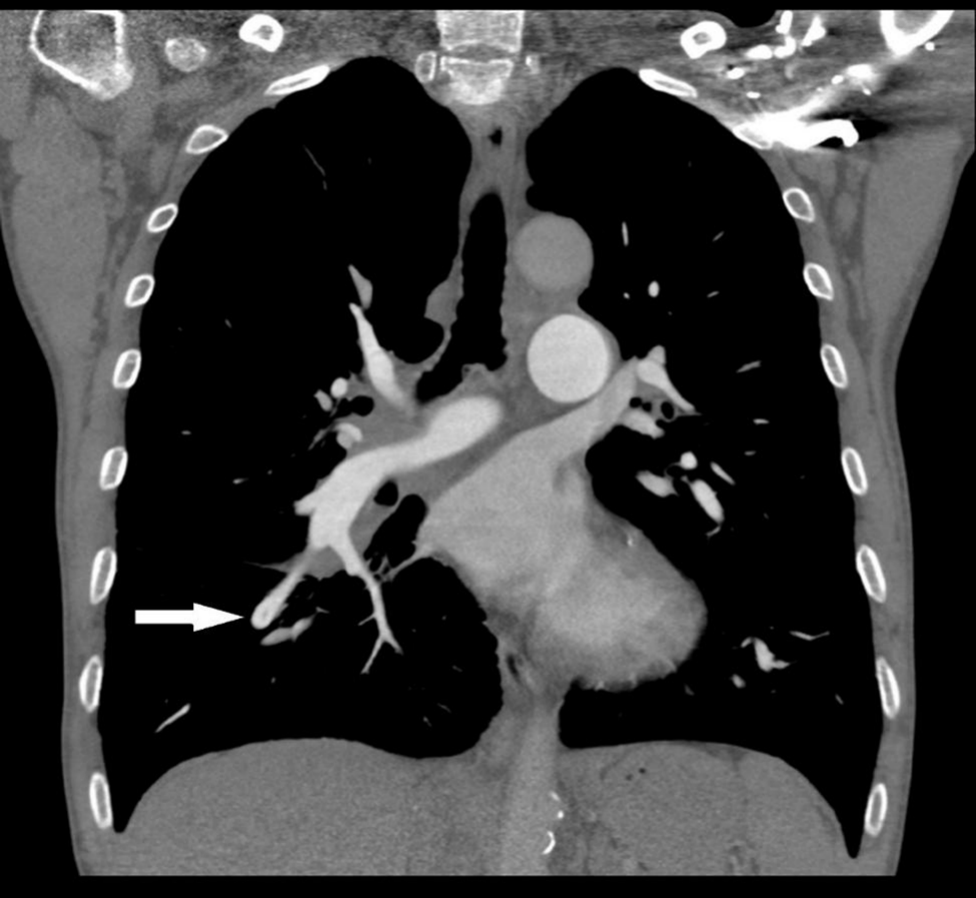
The examination of CT scan.


A 62-year-old male patient was admitted to our hospital with chief complaints of progressive chest tightness and suffocation that had persisted for 2 months. These symptoms rapidly worsened within 3 days. Upon admission, the patient underwent routine blood test: the white blood cell count was 5.46 × 10^9^/L, the red blood cell count was 3.90 × 10^12^/L, the hemoglobin (Hb) level was 98 g/L, and the platelet count was 372 × 10^9^/L. D-Dimer was measured at 3,250 ng/mL, while the arterial blood gas analysis revealed a oxygen partial pressure of 65 mmHg. The electrocardiogram displayed changes in ST-T of multiple leads, and lung function tests demonstrated moderate-to-severe obstructive ventilation dysfunction with a positive bronchiectasis test. Following computed tomography (CT) examination (Figure 1), the patient was diagnosed with acutely exacerbated chronic obstructive pulmonary disease and a medium-risk right pulmonary embolism. The patient responded well to a 2-week treatment of 1250IU ih q12h low-molecular-weight heparin anticoagulant therapy. The patient’s medication was switched to Xarelto 15 mg orally twice daily and gradually reduced until discontinued after 6 months. However, after 2 weeks of treatment, the patient’s Hb level progressively decreased, while the levels of globulin and β2-microglobulin significantly increased.

**Informed consent:** Informed consent has been obtained from all individuals included in this study.
**Ethical approval:** The research related to human use has been complied with all the relevant national regulations, institutional policies and in accordance with the tenets of the Helsinki Declaration, and has been approved by the authors’ institutional review board or equivalent committee.

### 2.2 Auxiliary examination

**Figure 2:**
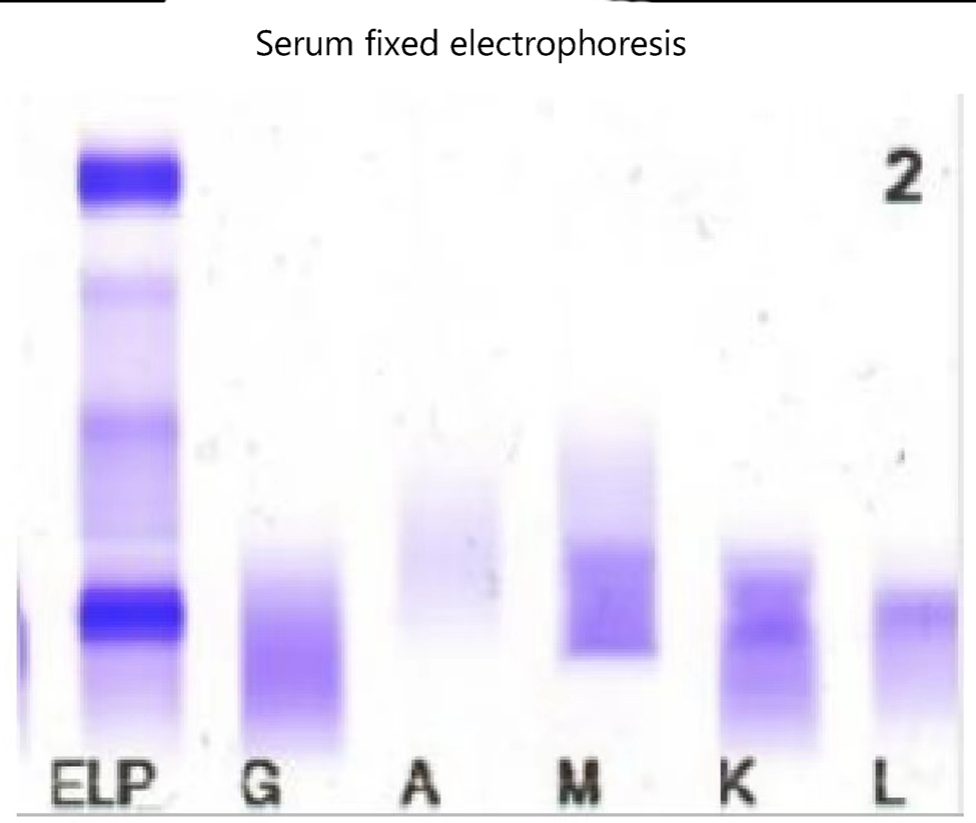
Serum immunofixation electrophoresis.

**Figure 3:**
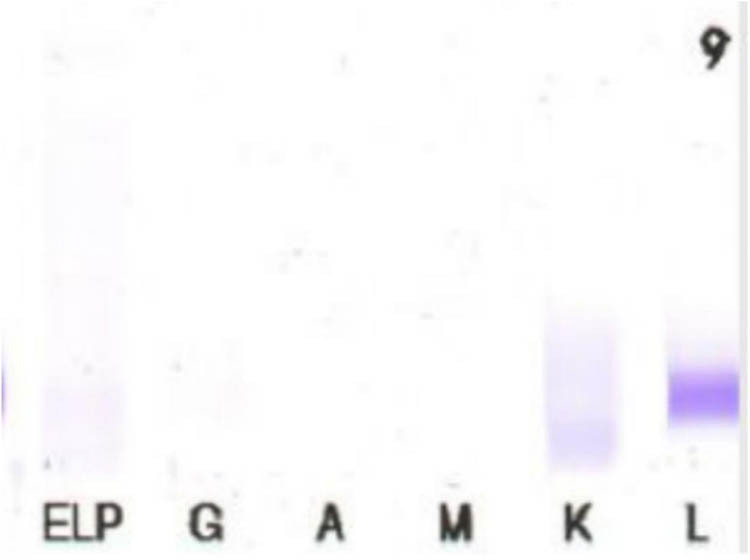
Urine immunofixation electrophoresis.

**Figure 4:**
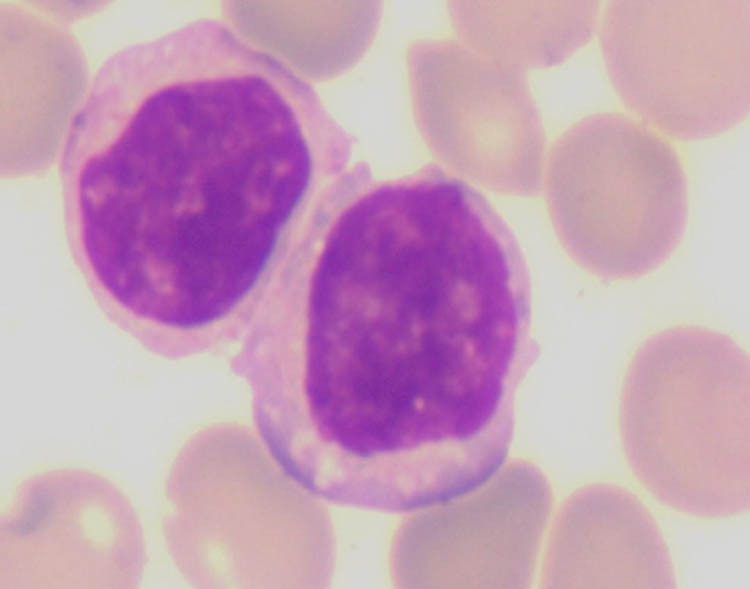
Bone marrow smears.

**Figure 5:**
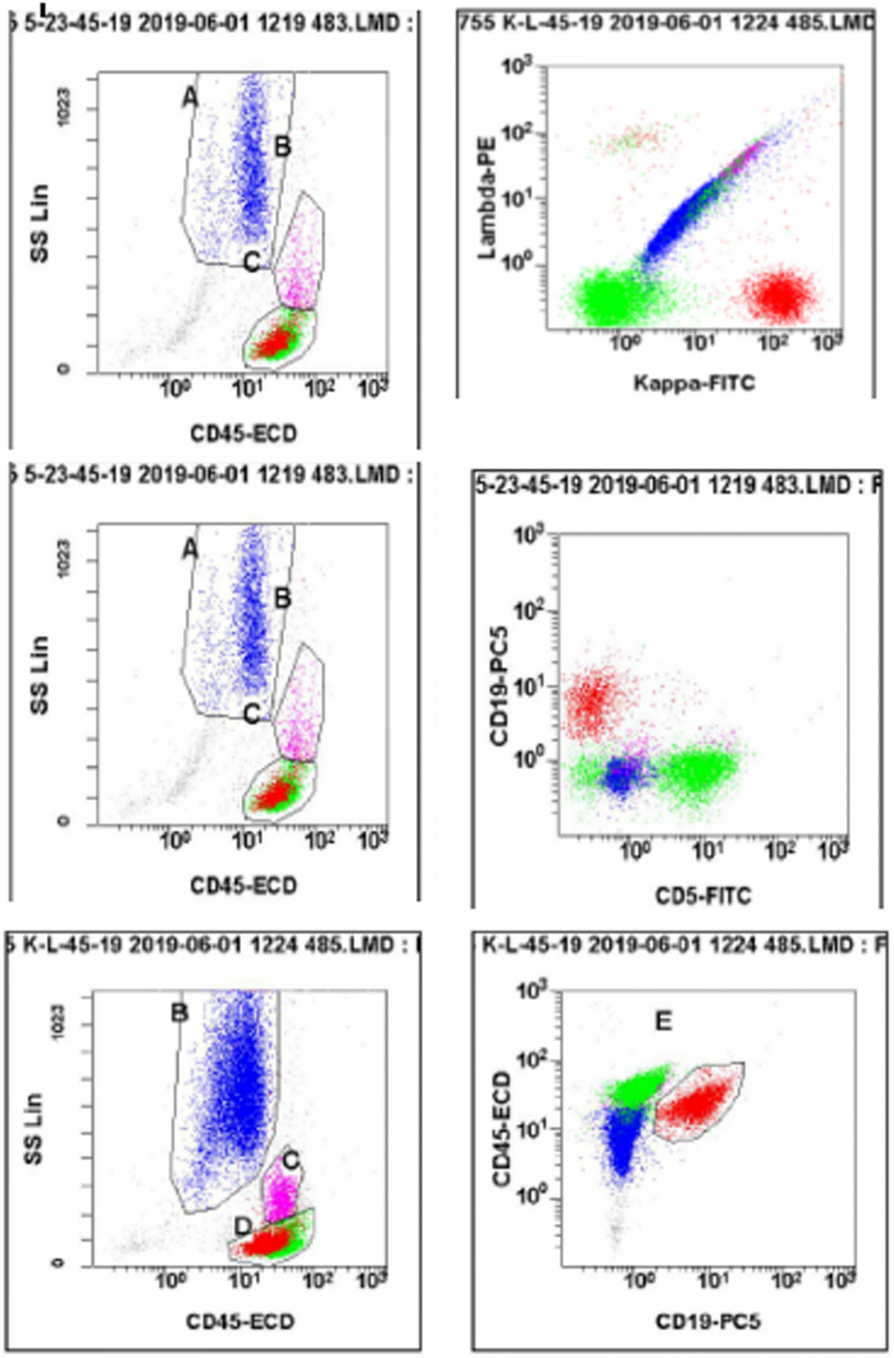
Flow cytometry analysis.

After a 2-week period, investigators noticed an unusual band indicating the presence of M protein during protein electrophoresis. The quantitative analysis of the serum immunoglobulin revealed that the serum levels of immunoglobulin G, immunoglobulin A, IgM, and light chain κ and λ values were 12.7, 1.3, 5.6, 8.54, and 2.47 g/L, respectively. Agarose gel electrophoresis was used to perform serum immunofixation electrophoresis (IFE) (Figure 2), which indicated the presence of a monoclonal IgM κ component and a monoclonal IgM λ component in the gamma region. The urine IFE (Figure 3) exhibited only a monoclonal λ component. Additionally, no hepatosplenomegaly was found via B-ultrasound. Examination of the bone marrow smears (Figure 4) showed predominantly abnormal lymphocytes, alongside morphologically normal plasma cells, accounting for 4.5% of the nucleated hematopoietic cells. Hematoxylin and eosin (H&E) and periodic acid-Schiff staining of the bone marrow biopsy showed an extremely hyperplastic marrow (approximately 90%) and abnormal cell proliferation. Flow cytometry analysis (Figure 5) indicated that these cells were positive for cKappa, CD38, CD138, CD56, and CD81, but negative for CD117, CD19, CD27, and cLambda. Immunohistochemical staining (IHCS) revealed that these cells were positive for CD20, PAX5, CD138, Kappa, Lambda, and CD56, but negative for CD3, CD5, CD10, and CyclinD1. No skeletal lesions were found. Additionally, the gene tests for MyD88 mutation showed a positive result, and CXCR4 gene test showed a negative result. Enlarged and fused lymph nodes were observed in the neck, axilla, abdominal cavity, retroperitoneum, and groin through CT scan and ultrasonic inspection. Lymph node biopsy analysis by IHCS showed that the cells were positive for CD20, Pax5, CD38, CD138, CD43, Bcl2, and MUM, but negative for CD3, CD5, CD10, CD23, CD21, and CyclinD1. Ki67 was expressed in 15% of the nucleated cells. The IgH rearrangement tests revealed positive results for IgHA, D, and IgK-A, while TCR rearrangement tests were negative.

### 2.3 Clinical diagnosis

According to the aforementioned information, the patient was diagnosed with IgM-k/L double-clonal LPL/WM, in due course.

### 2.4 Clinical treatment

Following the diagnosis, the patient underwent eight cycles of R-CHOP treatment and exhibited a positive therapeutic response. The levels of M protein in the serum decreased from 64.7 to 0, IgM from 50.4 to 9.1 g/L, β2 MG from 3.15 to 1.61 mg/L, and Hb increased from 95  to 136 g/L.

### 2.5 Clinical follow-up

At the time this article was completed, the patient had been in remission for 3 months. Ongoing follow-up will be conducted by the investigators to monitor the progress of the patient.

## 3 Discussion

LPL/WM is a unique type of lymphoproliferative disease that is identified by the infiltration of plasmacytoid lymphocytes in the bone marrow and a significant elevation in monoclonal IgM serum levels. This subset of lymphoproliferative diseases is categorized as a small B-cell indolent lymphoma with plasma cell differentiation and accounts for less than 2% of all NHLs [1].

The diagnostic criteria for WM include (1) the detection of monoclonal IgM in patient’s serum; (2) the invasion of plasma cell-like or plasma cell-differentiated small lymphocytes into the trabecular space of the bone marrow immunophenotypes: CD19 (+), CD20 (+), sIgM (+), CD22 (+), CD25 (+), CD27 (+), FMC7 (+), CD5 (+/−), CD10 (−), CD23 (−), and CD103 (−). Approximately 10–20% of patients can partially express CD5, CD10, or CD23; and (3) exclusion of other known types of lymphoma. Additionally, the MYD88 L265P mutation is present in 90% of the WM cases but cannot be used as the sole standard for the diagnosis of WM as it is also present in other small B-cell lymphoma, diffuse large B-cell lymphoma, etc. In this specific case, monoclonal IgM was detected in the patient’s serum, bone marrow biopsy, and lymph node biopsy; blood and urine IFE results were consistent with the diagnosis of WM.

About 30% of the patients with WM are asymptomatic [2]. The symptoms of WM varied and are complex [3]. The most commonly reported symptoms are anemia, dizziness, fatigue, and shortness of breath. Conversely, the increase in serum level of monoclonal IgM could lead to hyperviscosity [4], cryoglobulinemia, cold agglutinin syndrome, coagulation abnormal function, peripheral neuropathy, rarely amyloidosis, and other organ disorders. However, the pulmonary embolism is an uncommon complication in patients with WM.

According to a study, [5] the thrombotic state of patients with monoclonal gammaglobulinemia is complex and involves multiple factors: (1) malignant tumor-related factors, including an increase in paraprotein content, the release of inflammatory cytokines (such as IL-6), and changes in blood coagulation, which can potentially lead to hyperviscosity syndrome; (2) patient-related, such as the presence of central venous catheter, hypoalbuminemia, renal failure, immobilization, and obesity; and (3) treatment-related factors, including the use of immunomodulatory drugs during the treatment, which may promote thrombosis. In this particular case, malignant tumor-related factors were the primary contributing factors to the thrombotic state observed in the patient.

An elevation in the serum level of IgM in patients with WM is frequently monoclonal, although it can sometimes be polyclonal. To date, no large-scale reports and systemic studies have documented double-clonal WM. Double-cloned immunoglobulin refers to the presence of two distinct abnormal bands on the IFE, both of which originate from the same B-cell clone that has split into two clones due to antigen selection or malignant transformation [6]. The phenomenon of double-clonal immunoglobulin remains poorly understood. Currently, only a few studies support the possibility that a single malignantly transformed B cell may generate double-clonal immunoglobulin [7,8,9]. Earlier studies have suggested that immunoglobulin and gene rearrangement typically exhibit the same heavy chain and the same light chain. Based on this observation, the two distinct light chains identified in this study may have originated from double clones during the process of malignant transformation.

Biclonal WM is a relatively uncommon occurrence. Some experts suggest that a patients’ clinical symptoms may be determined by the type of monoclonal immunoglobulin with the highest concentration, while others propose that the concentration of monoclonal immunoglobulin may not be significantly correlated with clinical manifestations. A study on monoclonal immunoglobulin LPL found that patients’ primary clinical manifestations were anemia and fatigue [10]. According to Mullikin et al., the clinical characteristics, treatment response, and prognosis of patients with biclonal immunoglobulinemia do not appear to be different from those of typical LPL [11].

Limitations: This study has several limitations. First, it is a case report and involved only one patient resulting in a limited sample size. Second, the clinical follow-up period was relatively short, and it is crucial to observe the long-term prognosis of the patient.

This study reports on a rare lymphoproliferative disease and its rare complication. However, due to its extremely low incidence, the pathophysiology, prognosis, and optimal treatment have not been extensively studied. It is unclear whether double-cloned WM is prone to transform to progressive lymphoproliferative diseases. Braggio reported a case in which double-clonal expression of WM progressed to multiple myeloma in a later stage [2].

## 4 Conclusion

The patient in this case report initially presented with pulmonary embolism, which responded well to anticoagulation therapy. However, during the follow-up, a gradual decrease in Hb and a significant increase in globulin level indicated that the underlying disease was WM. The case highlights the importance of vigilant follow-up for patients with unexplained pulmonary embolism to rule out rare causes. Although the patient showed a positive therapeutic response, the long-term clinical prognosis requires further monitoring.
